# Prevalence of reduced visual acuity among adolescents in Jiaocheng County, Shanxi Province

**DOI:** 10.1186/s12886-022-02567-9

**Published:** 2022-08-16

**Authors:** Yang Yang, Chunhui Li, Yan Gao

**Affiliations:** 1 Shanxi Medical University, Taiyuan, Shanxi Province China; 2 Department of Ophthalmology, the First Hospital of Shanxi Medical University, Taiyuan, 030001 Shanxi Province China; 3 Shanxi Eye Hospital, Taiyuan, 030001 Shanxi Province China

**Keywords:** Reduced visual acuity, Myopia, Adolescents

## Abstract

**Purpose:**

To investigate the prevalence of reduced visual acuity among adolescents in Jiaocheng County, Shanxi Province.

**Methods:**

Fourteen thousand fifty-one Jiaocheng County students aged 7 to 21 were chosen to engage in this research project in 2019. For uncorrected distance visual acuity (UCDVA) testing, a 5 m standard logarithmic visual sharpness E chart was utilized, and for diopter examination of those with reduced UCDVA, computerized optometry was used. The factors linked to reduced UCDVA in students were investigated using logistic regression analysis.

**Results:**

In Jiaocheng County, Shanxi Province, the prevalence of reduced UCDVA among adolescents was 77.54% in 2019, with the highest rate of severely reduced UCDVA at 47.58% and myopia accounting for the highest proportion of reduced UCDVA, with myopia rates above 90% in all age groups. Girls, those who live in counties, those who are anxious about their studies, and those who dedicate more time to schoolwork are more prone to suffer from a decrease in UCDVA; those who spend more time outdoors and get adequate sleep are less likely to have reduced UCDVA, according to logistic regression analysis.

**Conclusion:**

Adolescents in Jiaocheng County, Shanxi Province, have a high prevalence of reduced UCDVA, and interventions targeting key populations should be increased based on effective prevention and control of reduced UCDVA among local adolescents.

## Introduction

The number of persons with reduced UCDVA has risen year after year, posing a serious threat to young people’s health and becoming a significant public health problem globally [1]. Myopia is the most frequent kind of reduced UCDVA [2]. Some estimates predict that by 2050, the number of persons with myopia will have increased to 5 billion, accounting for over half of the global population [3]. Myopia impacts young people’s academics, social lives, future employment prospects, and physical and emotional health. High myopia can cause irreversible vision loss due to retinal detachment, macular retinoschisis, and glaucoma [4, 5]. According to the literature, myopia rates in Asia are much greater than in other world regions, with high school pupils having roughly 90% myopia and 20% high myopia [6]. This study analyzed the reduced UCDVA of teenagers in Jiaocheng County, Shanxi Province, to create a scientific foundation for developing strategies and treatments to prevent and treat reduced UCDVA in students.

## Subjects and methods

### Subjects

Cross-sectional epidemiology research underpins this project. In 2019(February 1--December 31), 14,051 students aged 7 to 21(13.47 ± 3.13) from the county and suburban primary, middle, and high schools volunteered to participate in this survey research. Our researchers examine pupils in one school before moving on to the following one. Inclusion and exclusion criteria: Inclusion criteria: Age: 7–21 years old； High degree of cooperation and compliance from students during all tests. Exclusion criteria: Excluding students with keratoconus within 3 days； Excluding students with organic eye disease.

### Methodology

A 5 m standard logarithmic visual acuity scale was used to assess students’ uncorrected distance visual acuity. The diagnostic criteria for reduced UCDVA were:≥5.0 as normal, ≤4.8–4.9 as mildly reduced UCDVA, 4.6–4.7 as moderately reduced UCDVA, and ≤ 4.5 as severely reduced UCDVA. The diopter examination with a computerized optometer and myopia was diagnosed as SER ≤ − 0.50 D, hyperopia: SER> + 0.75D. Other types of reduced UCDVA are classified as other.

Students were asked to fill out questionnaires on whether they felt pressured to study, how much time they spent on homework each day, how much time they spent resting each day, and how much time they spent outside.

### Ethical approval

The Ethics Committee of The People’s Hospital of Jiaocheng County, Shanxi Province, gave their approval to this study. We also got the permission of all of the pupils and their guardians.

### Statistical analysis

The data were analyzed using the statistical application SPSS 26.0. The chi-square test was used to look for differences among groups. The factors connected to reduced UCDVA in students were investigated using logistic regression, with *P*<0.05 as a statistically significant difference.

## Results

1. The overall prevalence of reduced UCDVA detection in a county in Shanxi Province

**Fig. 1:**
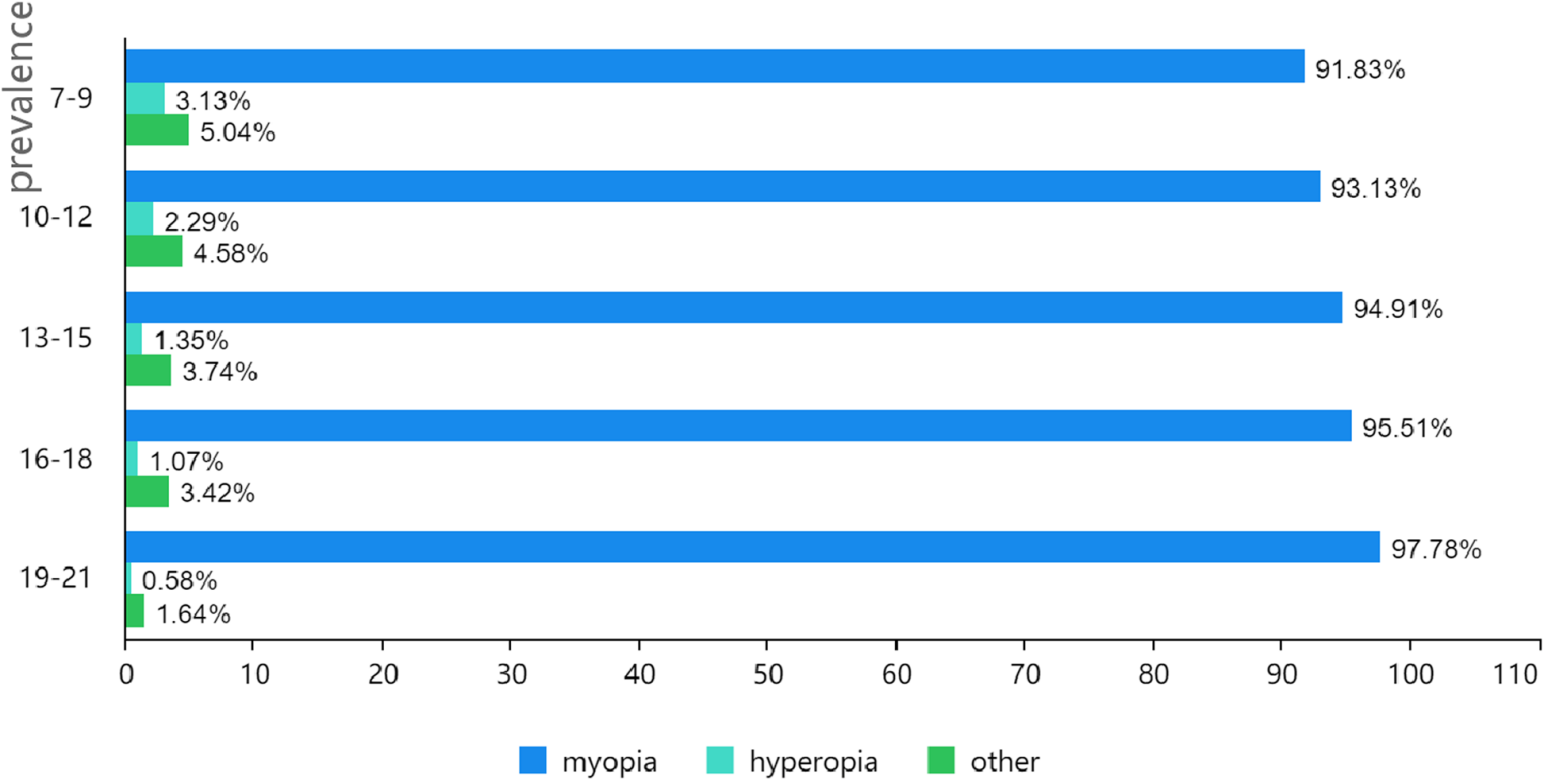
The prevalence of myopia, hyperopia, and other among reduced UCDVA by age group

**Fig. 2:**
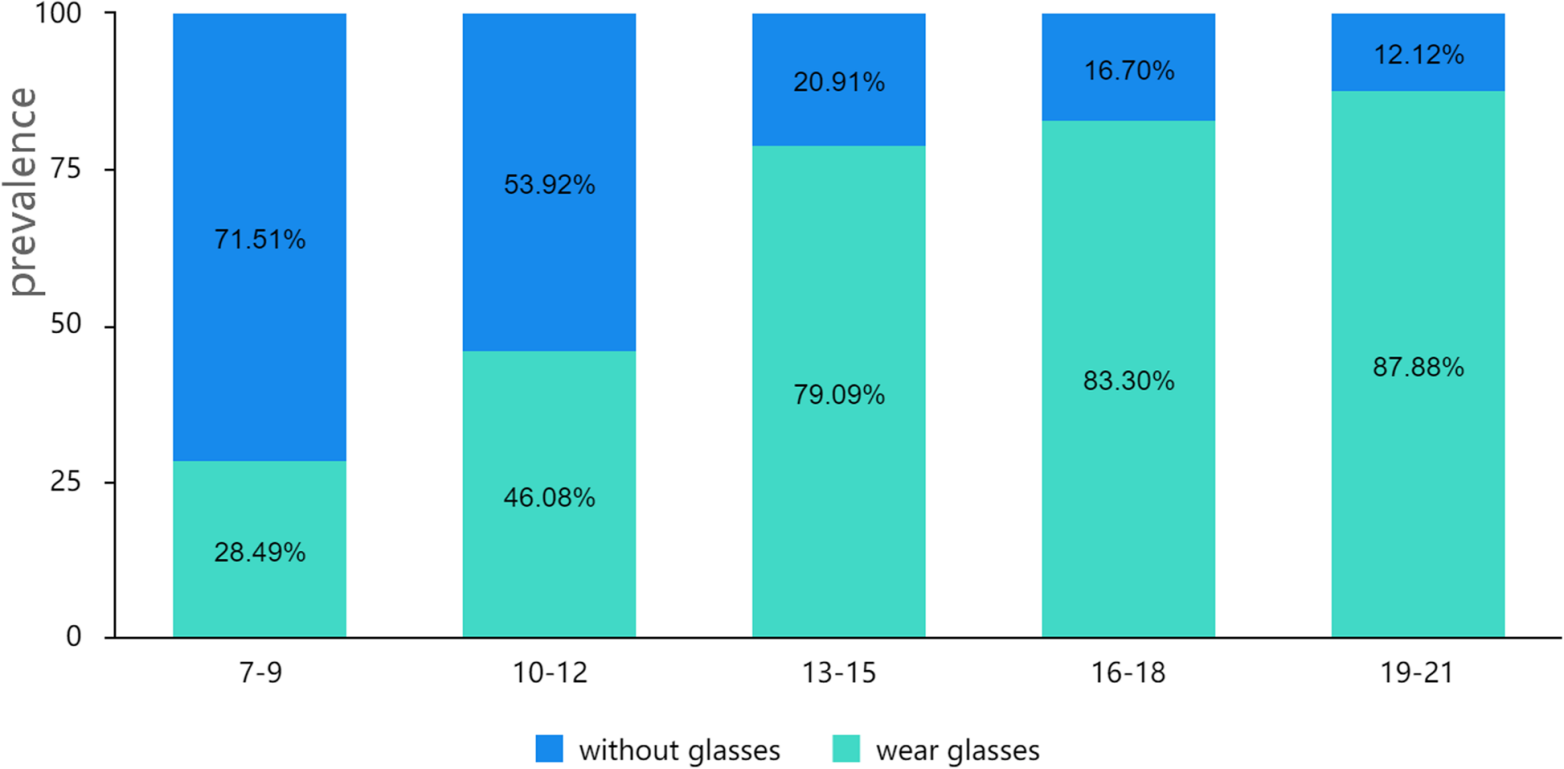
The prevalence of myopia wearing glasses by age group

The number of adolescents with reduced UCDVA in Jiaocheng County, Shanxi Province, investigated in this study was 10,895, and the detection rate of reduced UCDVA was 77.54%. Myopia accounted for the highest proportion of reduced UCDVA, with myopia rates above 90% across all age ranges. Myopia wear also accelerates with aging. The highest prevalence of reduced UCDVA among county girls and the lowest prevalence of reduced UCDVA among suburban boys were found in all age groups, with 87.43 and 67.82%, respectively. (Table 1, Figs. 1 and 2).

**Table 1:** The prevalence of reduced UCDVA among students in Jiaocheng County, Shanxi Province

Age group /years	County girls		County boys		Suburban girls		Suburban boys		Girls		Boys		Total	
	n %		n %		n %		n %		n %		n %		n %	
7–9	154	60.39	202	58.38	196	53.85	182	39.82	350	56.54	384	47.82	734	51.62
10–12	502	76.64	547	65.12	1003	69.60	963	61.89	1505	71.80	1510	63.02	3015	67.12
13–15	1044	91.26	1092	83.81	832	86.76	802	77.19	1876	89.21	1894	80.87	3770	84.81
16–18	818	95.34	636	92.31	415	90.61	469	82.43	1233	93.69	1105	87.84	2338	90.83
19–21	417	96.08	357	92.25	136	94.44	128	83.66	553	95.67	485	89.81	1038	92.84
Total	2935	87.43	2834	78.98	2582	76.96	2544	67.82	5517	82.20	5378	73.28	10,895	77.54

2 Characteristics of changes in reduced UCDVA among adolescents in Jiaocheng County

Reduced UCDVA remained high among adolescents of all ages. The detection rate of reduced UCDVA increased gradually with age (x^2^ = 3415.598, *p* < 0.01), as did the rate of severely reduced UCDVA, with the highest rate of severely reduced UCDVA among girls in county areas and the lowest rate among boys in suburban areas, with probabilities of 60.77 and 36.34% respectively. (Table 2).

**Table 2:** The prevalence of reduced UCDVA by county/suburb, gender, and age group

Age group /years	n	County girls/%			County boys /%			Suburban girls/%			Suburban boys/%			Total/%		
		Mild	Moderate	Severe	Mild	Moderate	Severe	Mild	Moderate	Severe	Mild	Moderate	Severe	Mild	Moderate	Severe
7–9	1422	43.14	9.80	7.45	37.86	13.29	7.23	40.66	7.42	5.77	27.13	6.56	6.13	36.08	9.00	6.54
10–12	4492	31.15	17.10	28.40	24.64	14.64	25.83	27.00	13.60	29.01	26.03	11.89	23.97	26.83	13.71	26.58
13–15	4445	14.51	10.75	66.00	15.43	12.89	55.49	13.66	9.38	63.71	14.63	12.32	50.24	14.62	11.45	58.74
16–18	2574	5.87	6.33	81.93	8.15	8.00	73.17	12.30	7.38	73.15	15.57	9.71	60.62	9.67	7.69	73.47
19–21	1118	5.07	6.22	84.79	6.20	8.27	77.78	9.03	3.47	81.94	6.54	5.88	71.24	6.17	6.53	80.14
Total	14,051	16.47	10.19	60.77	17.31	11.87	49.80	21.94	10.46	44.56	20.69	10.88	36.34	19.12	10.85	47.58
X^2^_linear by liner_		759.19			657.30			655.66			586.78			2896.69		
p		<0.01			<0.01			<0.01			<0.01			<0.01		

Multi-factor analysis of reduced UCDVA

Logistic regression analysis was conducted with gender, county, suburb, study pressure, homework, outdoor activity, and sleep time as independent variables, with reduced UCDVA as the dependent variable (0 = normal, 1 = reduced UCDVA) and stratifying by age group. It discovered that girls were more inclined to have reduced UCDVA than boys in all age groups and that county students were more prone to have reduced UCDVA than suburban students. Those who were stressed about their studies were more inclined to have reduced UCDVA; those who consumed more than 2 hours on homework were more inclined to have reduced UCDVA; those who consumed more than 2 hours outdoors were less prone to have reduced UCDVA, and those who got enough sleep were less prone to have reduced UCDVA. (Table 3).

**Table 3:** Logistic regression models predicting reduced UCDVA among students

Variable	7–9 years old	10–12 years old	13–15 years old	16–18 years old	19–21 years old
Gender	0.70 (0.57–0.87)	0.67 (0.59–0.76)	0.51 (0.43–0.60)	0.48 (0.36–0.64)	0.39 (0.24–0.65)
County/Suburb	1.70 (1.37–2.10)	1.23 (1.07–1.41)	1.53 (1.29–1.80)	1.41 (1.07–1.84)	2.05 (1.29–3.28)
Feeling learning pressure	1.24 (1.00–1.54)	1.58 (1.39–1.78)	1.43 (1.22–1.69)	1.36 (1.03–1.78)	0.56 (0.34–0.93)
Time of homework every day≥2 h	1.40 (1.14–1.73)	1.58 (1.40–1.80)	1.31 (1.10–1.54)	1.52 (1.16–1.99)	1.81 (1.14–2.89)
Outdoor time every day≥2 h	0.54 (0.43–0.66)	0.70 (0.62–0.79)	0.77 (0.64–0.90)	0.61 (0.46–0.80)	0.43 (0.27–0.68)
Sleep time every day	0.78 (0.62–0.95)	0.75 (0.66–0.85)	0.83 (0.70–0.97)	0.73 (0.56–0.96)	0.58 (0.36–0.95)

Gender:0 = girls，1 = boys； County/Suburb:0 = Suburb，1 = County； Feeling learning pressure:0 = Feeling not learning pressure，1 = Feeling learning pressure； Time of homework every day:0 = less than 2 h;1 = more than 2 h; Outdoor time every day: 0 = less than 2 h;1 = more than 2 h; Sleep time every day: 0 = not enough sleep, 1 = enough sleep (7-9 years ≥ 10 h, 10-12 years ≥ 9 h，13-21 years ≥ 8 h)

## Discussion

The detection rate of reduced UCDVA among adolescents in Jiaocheng County, Shanxi Province, was 77.54% in 2019, higher than the national rate of reduced UCDVA among adolescents in 2014 [7]. The rate of severely reduced UCDVA was the highest, indicating that the situation of reduced UCDVA is complicated and must be addressed. Myopia was the most common cause of reduced UCDVA, with rates above 90% in all age categories, with the frequency of myopia increasing with age.

We found that reduced UCDVA is more common in county regions than in suburban areas, and it is more prevalent in girls than in boys. It is the same as most research [8, 9]. According to some research, people in cities are more likely to develop myopia, probably due to a crowded environment, a stronger emphasis on education, and more close-range activities [10]. Myopia prevalence is also related to gender, with girls developing earlier in adolescence and hormonal changes that may contribute to myopia [11], as well as boys and girls having different personalities, with girls preferring quiet indoor activities, studying harder, spending more time close and less time outdoors, and having a higher prevalence of myopia. In addition, myopia wear is common and gets worse as people age.

Outdoor activities, study time, education system, close work, and proper sleep have all been linked to the development of myopia [12–14]. Girls, county regions, longer homework time, and high school stress were also identified to be risk factors for reduced UCDVA, while proper sleep and more time spent outside were found to be protective factors. According to some research, people who work at close ranges are more likely to be myopic, and children who use their eyes more closely or live in crowded situations are more likely to be myopic than their peers [15]. Myopia was shown to be more common in students who studied for more than 5 h each day, according to Mavracanas et al. [16] Myopia is more prevalent in terms of close reading, according to Ip et al. [17] Myopia is substantially more common in Singapore, Korea, and China than in other nations, presumably due to their high-pressure education systems [18], which require students to study intensively and under stress, increasing the frequency of myopia. Dirani et al. detected a relationship between myopia and outdoor activities in youngsters [19]. The more outdoor exercise was related to a reduced prevalence of myopia, according to Wu et al. [20] The consequences of our study are consistent with these conclusions.

## Conclusion

Even though our study has some limitations, such as the lack of dilated pupils to paralyze the ciliary muscle and the lack of sufficient evidence for myopia diagnosis, it still offers some scientific support for the prevention and control of myopia and reduced UCDVA among adolescents in Shanxi Province.

In conclusion, this study examines the current state of reduced UCDVA among adolescents in Shanxi Province’s Jiaocheng County, concluding that the rate of reduced UCDVA is significant. It is proposed that the government prioritize preventing and controlling reduced UCDVA among girls in urban areas, starting with younger students by focusing on prevention and control of reduced UCDVA, establishing good eye care and healthy eye use habits from an early age, reducing homework, strengthening outdoor exercise, ensuring adequate sleep, regularly monitoring visual acuity, and establishing graphic acuity standards.

## Data Availability

The datasets analysed in this study are available from the corresponding author (Chunhui, Li, lchgyx@163.com) upon reasonable request.

## Abbreviations

UCDVA: uncorrected distance visual acuity
